# Changing Clinical Spectrum and Disease Progression in Young Patients With Bladder Cancer: A Retrospective Observational Study

**DOI:** 10.7759/cureus.82516

**Published:** 2025-04-18

**Authors:** Mehul Agarwal, Vikas Kumar Panwar, Ankur Mittal, Dharam Dev, Siddharta Saxena, Harshit Agrawal, Avin Singhal, Nalin Srivastava, Kunal Malhotra

**Affiliations:** 1 Urology, All India Institute of Medical Sciences, Rishikesh, Rishikesh, IND

**Keywords:** bladder cancer, disease progression, recurrence, survival, urothelial carcinoma, young patients

## Abstract

Background

Bladder cancer is a rare entity in young adults, and the course of the disease is not well defined. This study aims to analyze the clinical characteristics, risk factors, disease progression, and survival outcomes of bladder cancer patients aged 40 years or younger.

Methods

A retrospective review was conducted of 162 young bladder cancer patients treated from August 2017 to May 2024. Recurrence-free survival and overall survival were analyzed using Kaplan-Meier analysis.

Results

Of the 162 patients, 138 (85.2%) were male, with a mean age of 34.5 years. Key risk factors included tobacco use (80 patients, 49.4%) and occupational exposure to chemicals (50 patients, 30.9%). The predominant histology was urothelial carcinoma (159 patients, 98.1%). Muscle-invasive bladder cancer (MIBC) was observed in 42 patients (26.4%). Among patients with non-muscle-invasive bladder cancer (NMIBC), 61 (53.5%) had high-grade tumors. High-grade tumors and recurrence were frequent, with a mean recurrence-free survival of 3.7 years. Patients with muscle-invasive disease had poorer survival (mean 3.4 years) compared to those with non-muscle-invasive disease (mean 6 years).

Conclusion

Young bladder cancer patients exhibit a more aggressive disease course than reported in older cohorts. The findings underscore the importance of early detection, targeted treatment strategies, and tailored surveillance in young patients due to their unique risk factors and disease characteristics.

## Introduction

Urothelial carcinoma is the tenth most common cancer overall and the sixth most common malignancy in men worldwide [1]. The global incidence rate (per 100,000 person-years) is 9.5 in men and 2.4 in women [2]. In India, each year, 22,548 people are diagnosed with bladder cancer, and approximately 12,353 patients die from the disease. The global age-standardized mortality rate (per 100,000 person-years) is 3.3 for men and 0.86 for women [1]. The median age at diagnosis of bladder cancer is 71 years for females and 69 years for males. Urinary bladder cancer (UBC) is rare in individuals under 40 years of age, with an incidence reported between 1.35% and 1.6% in some studies. Among patients younger than 20 years, the incidence drops further to just 0.003%. Due to its rarity, the natural history and prognosis of UBC in these age groups remain poorly understood [2].

The pathophysiology of bladder cancer in younger individuals has been a topic of interest, as it may differ from that in older populations. Studies have suggested that younger patients often present with lower-grade, non-muscle-invasive bladder cancer (NMIBC), which generally has a favorable prognosis [3]. However, a subset of young patients exhibit aggressive disease characteristics, including muscle-invasive bladder cancer (MIBC) and high-grade tumors, which significantly impact survival outcomes [4].

Given the paucity of data on young bladder cancer patients, this study aims to provide a comprehensive analysis of the clinical spectrum, risk factors, disease progression, and survival outcomes in individuals aged 40 years or younger. The main objectives of our study are to assess survival rates, recurrence, and progression in young patients with bladder cancer and to compare survival between NMIBC and MIBC.

By retrospectively reviewing patient data, we seek to bridge the knowledge gap and offer insights that may contribute to better management and tailored therapeutic strategies for young bladder cancer patients.

## Materials and methods

All patients diagnosed with urinary bladder carcinoma at or below 40 years of age at the institute from August 2017 to May 2024 were retrospectively analyzed. Data were collected from hospital records, including presenting complaints, comorbidities, presence of risk factors (including smoking and exposure to chemicals), ultrasonography of the kidney, ureter, and bladder (USG KUB) findings, contrast-enhanced computed tomography (CECT) chest with computed tomography (CT) urography, urine cytology, cystoscopy findings, histopathological examination (HPE) report, and management. Patients were followed up until June 1, 2024, for disease progression and recurrence. Patients with less than one month of follow-up were excluded from the study.

NMIBC patients either underwent regular follow-up, received intravesical therapy in the form of BCG, gemcitabine 2 g, or gemcitabine 1 g with docetaxel, or underwent upfront radical cystectomy according to European Association of Urology (EAU) guidelines. For NMIBC patients, initial treatment involved intravesical BCG therapy; however, due to periods of BCG shortage during the study, we adopted intravesical chemotherapy (gemcitabine and docetaxel) as an alternative, which is supported by the EAU guidelines [2]. Patients who received adequate intravesical therapy [2] were considered for the calculation of recurrence-free survival. NMIBC patients who underwent upfront cystectomy were classified as “very high risk” according to EAU risk stratification [2].

MIBC patients either underwent neoadjuvant chemotherapy in the form of dose-dense methotrexate, vinblastine, doxorubicin (Adriamycin), and cisplatin (ddMVAC); upfront radical cystectomy (cisplatin-ineligible); or bladder preservation protocol according to EAU guidelines [2]. Variant histology and metastatic disease received further management and were followed up and included in other categories for survival analysis.

Patient data regarding symptoms, disease, investigation, and treatment were accessible to all authors. Written informed consent was obtained from all patients, including consent for publication.

Since this was a retrospective analysis, approval was not required according to the institutional ethical committee. The principles of the Helsinki Declaration were followed. All authors confirm the availability of and access to the original data reported in this study.

Statistical analysis

Discrete categorical data, such as age, sex, comorbidities, risk factors, and tumor stage, were presented as frequencies and proportions. Continuous variables were described either as mean ± standard deviation or as median with interquartile range, depending on distribution. Categorical data were analyzed using the chi-square test or Fisher’s exact test, as appropriate, while means were compared using Student’s t-test or the Mann-Whitney U test. Statistical tests were conducted at a significance level of α = 0.05 (95% CI), using IBM SPSS Statistics for Windows, Version 23 (Released 2015; IBM Corp., Armonk, New York). Survival analysis was performed using the Kaplan-Meier method, with the log-rank test used to compare survival distributions among patient groups, accounting for censored observations.

## Results

Patient demographics

A total of 162 patients with carcinoma of the urinary bladder aged 40 years or younger were included in the study, of whom 138 were male (85.2%) and 24 were female (14.8%). The mean age was 34.53 years, with the youngest patient being 18 years old. Hematuria was the most common presenting symptom, occurring in 150 (92.6%) patients, with a mean duration of symptoms of three weeks.

A history of tobacco use was present in 80 (49.4%) patients, a history of alcoholism in 32 (19.7%) patients, and a history of occupational exposure to chemicals in 50 (30.9%) patients. A family history of malignancy was present in eight patients (5%). Among comorbidities, five patients (3%) were hypertensive. The various demographic characteristics of the patients are shown in Table 1.

**Table 1:** Demographic characteristics of patients

Variables	Values
Total patients	162
Mean age (years)	34.53
Sex (male/female)	138/24 (85.2%/14.8%)
Smoking status	80 (49.4%)
Family history of bladder cancer	8 (5%)
Comorbidities	5 (3%)

Histopathological diagnosis

Out of 162 patients, 159 had urothelial carcinoma, whereas three had variant histology (Table 2). Among the patients with variant histology of neuroendocrine tumor (NET), one patient received concurrent chemoradiotherapy (CTRT) and is on regular follow-up without recurrence, one received neoadjuvant chemotherapy and expired during the course of the disease, and one was lost to follow-up without receiving treatment.

**Table 2:** Histopathological diagnosis (post-TURBT) NET: neuroendocrine tumor, NMIBC: non-muscle-invasive bladder cancer, PUNLMP: papillary urothelial neoplasm of low malignant potential, TaLG: non-invasive low-grade papillary urothelial carcinoma, TaHG: non-invasive high-grade papillary urothelial carcinoma, T1LG: lamina propria-invasive low-grade urothelial carcinoma, T1HG: lamina propria-invasive high-grade urothelial carcinoma, MIBC: muscle-invasive bladder cancer, M1a: non-regional lymph node metastasis, M1b: distant organ metastasis.

Variables	Number of Patients (n=162)
Urothelial carcinoma	159(98.1%)
Neuroendocrine tumour (NET) (small cell carcinoma)	3 (1.9%)
NMIBC	114 (71.7%)
Papilloma	13 (11.4%)
PUNLMP	3 (2.6%)
TaLG	37 (32.5%)
TaHG	3 (2.6%)
T1LG	20 (17.5%)
T1HG	38 (33.3%)
MIBC	42 (26.4%)
Metastatic (M1a and M1b)	3 (1.9%)

Clinical outcomes and progression of NMIBC patients

**Figure 1:**
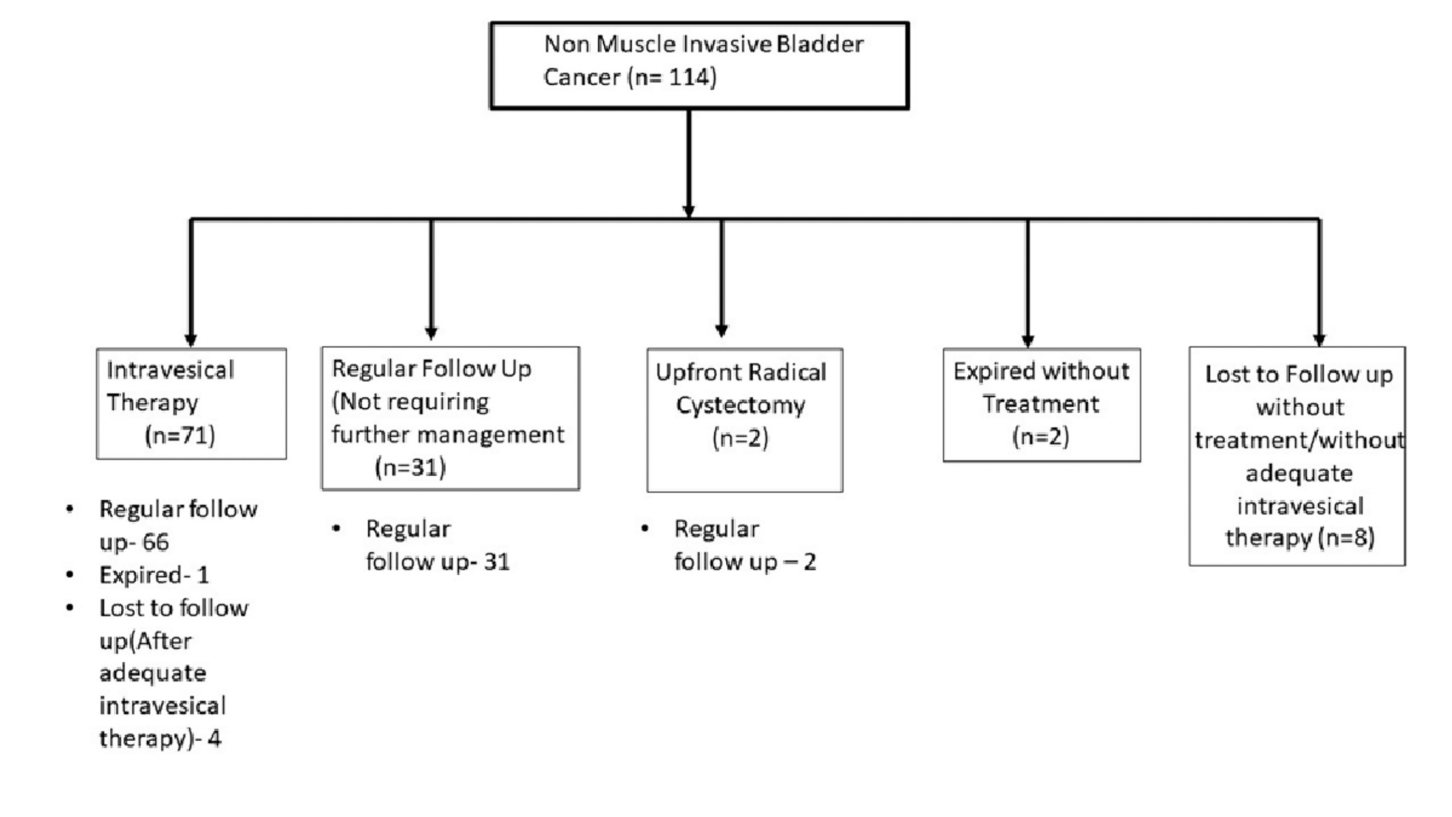
Non-muscle invasive bladder cancer (NMIBC) flow chart showing management and follow-up

Clinical management and outcomes of NMIBC patients are summarized in Figure 1.

**Figure 2:**
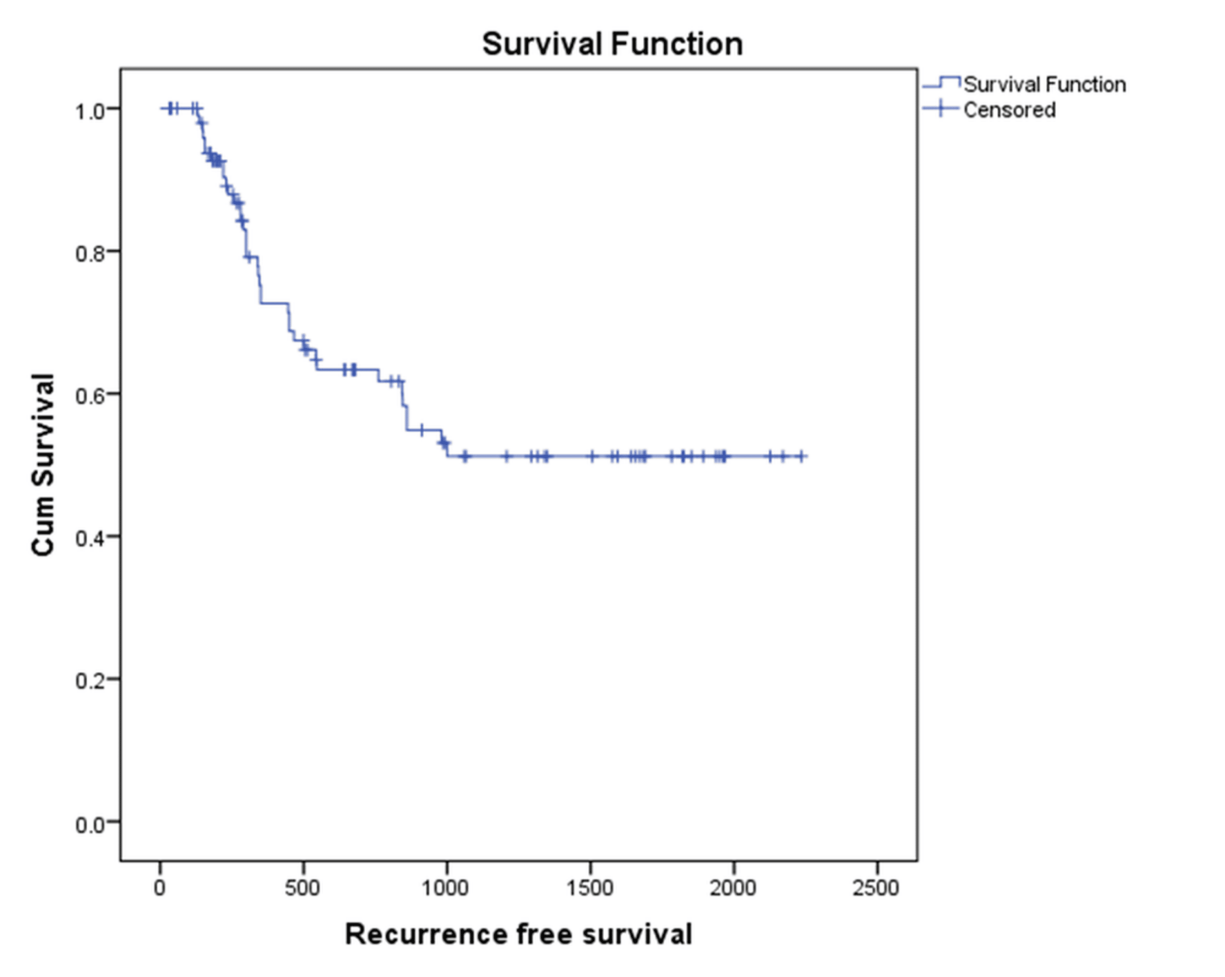
Kaplan-Meier curve showing recurrence free survival in NMIBC patients NMIBC: non-muscle-invasive bladder cancer, Cum: cumulative.

Out of a total of 114 patients with NMIBC, 102 patients who received adequate intravesical therapy or regular follow-up without cystoscopy were included for calculating recurrence-free survival. Thirty-seven (36%) patients experienced recurrence during follow-up and were managed accordingly. Mean recurrence-free survival was calculated as 3.7 years (3.2-4.3 years) in NMIBC patients (Figure 2).

Clinical outcomes and progression of MIBC patients

**Figure 3:**
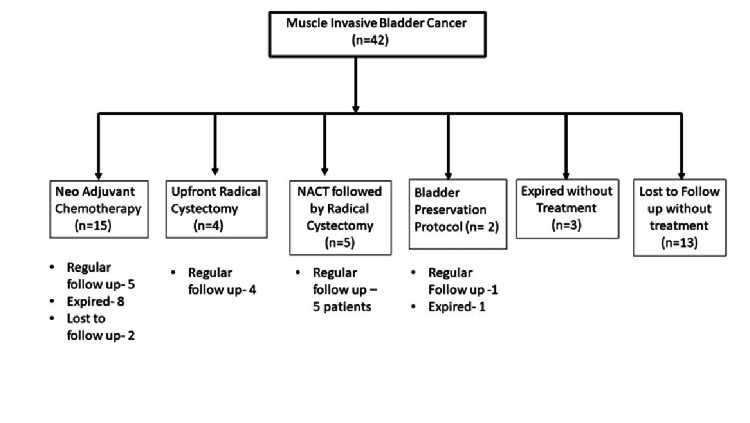
MIBC flow chart showing management and follow-up MIBC: muscle-invasive bladder cancer.

Around 42 (26.4%) patients had MIBC at diagnosis and were managed according to EUA guidelines after discussion in the Institutional Tumor Board. Clinical management and outcomes of MIBC patients are summarized in Figure 3.

Survival analysis

**Figure 4:**
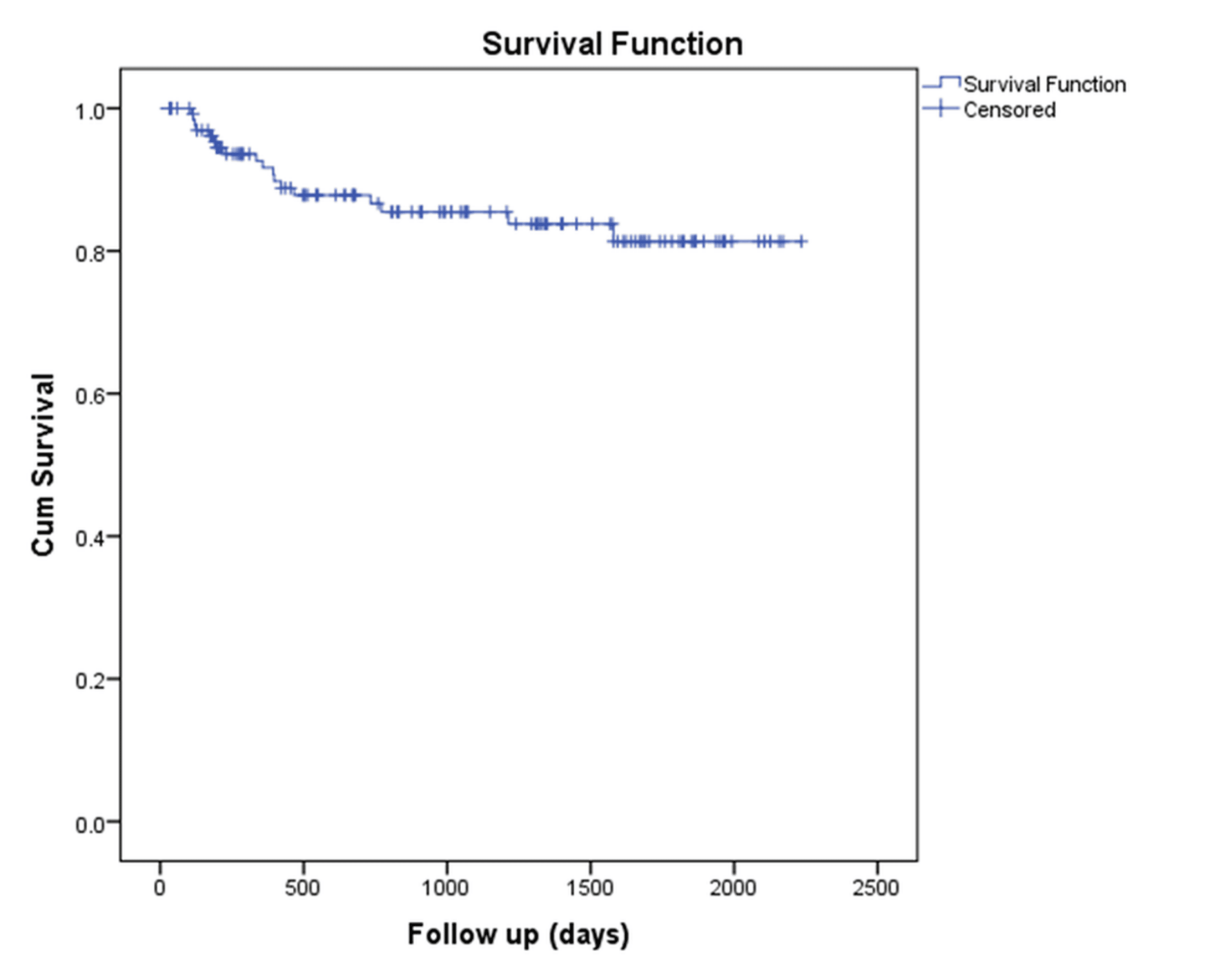
Kaplan-Meier curve showing overall survival in urinary bladder carcinoma patients Cum: cumulative.

The mean survival of young patients with urinary bladder carcinoma, as calculated from our study, was 5.3 years (4.9-5.6 years), with a 95% confidence interval (CI) (Figure 4).

**Figure 5:**
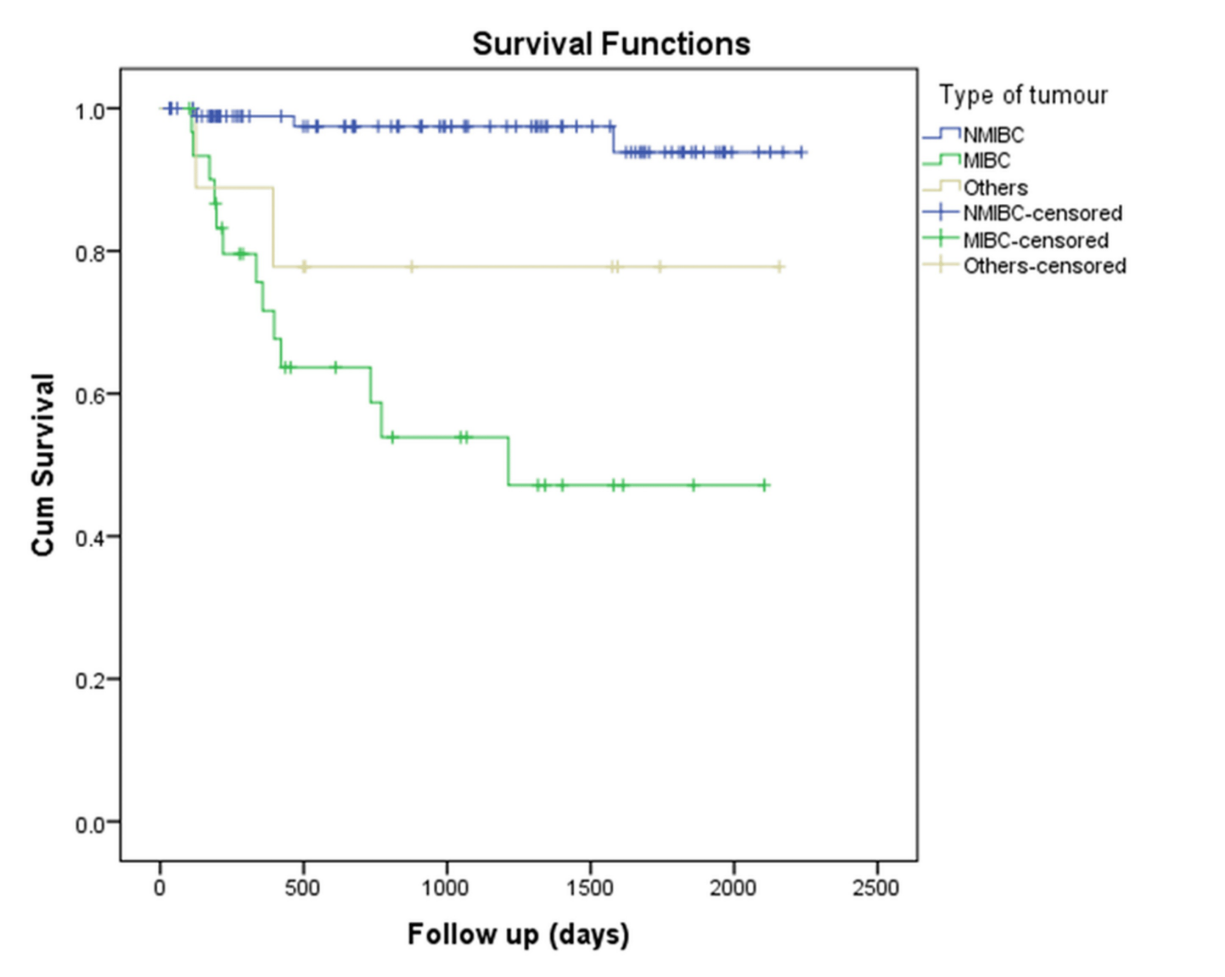
Kaplan-Meier survival curve comparing survival between different types of bladder cancers NMIBC: non-muscle-invasive bladder cancer, MIBC: muscle-invasive bladder cancer, Cum: cumulative.

The mean survival in NMIBC patients was calculated as 6 years (5.7-6.14 years), with a 95% CI, whereas the mean survival in MIBC patients was 3.4 years (2.5-4.3 years), with a 95% CI (Figure 5).

Three patients were diagnosed with metastatic urinary bladder carcinoma at presentation. Two patients expired during the course of palliative chemotherapy, and one patient was lost to follow-up.

## Discussion

Bladder cancer in young patients is rare, with most studies indicating a lower grade and a better prognosis compared to older individuals. Hematuria was the most common presenting symptom, occurring in 150 (92.6%) patients, with a mean duration of symptoms of three weeks. Most of the patients primarily presented to our center since it is the only government hospital in the region.

The most common cause is tobacco smoking, accounting for approximately 50% of cases, whereas occupational exposure accounts for 10% of all cases [5]. According to studies in the young population with urinary bladder carcinoma, 60.3% had a history of smoking and 29.3% had a history of occupational exposure, whereas in the elderly, these figures were 75.2% and 43.8%, respectively [6]. In our study, smoking and tobacco exposure was found in 49.4%, and occupational exposure was found in 30.9% of young patients with urinary bladder carcinoma. 

In histopathology, urothelial cancer represents the most common form, with more than 90% of bladder cancers being urothelial [7]. In our study, urothelial carcinoma was also the most common subtype among young patients, with a percentage of 98.1%.

Most past studies have shown that younger patients with bladder cancer tend to have low-grade, low-stage disease, with lower rates of progression and recurrence compared to older individuals. According to a study conducted by De la Calle et al., 90.3% of patients under 40 years of age were diagnosed with NMIBC and 9.7% with muscle-invasive disease, whereas in patients above 40 years of age, the proportions were 81.2% and 18.8%, respectively. In the same study, high-grade cancers were found in 27.3% of patients aged 18-40 years. This study concluded that younger patients typically present with non-muscle-invasive, low-grade tumors and have better survival outcomes compared to older patients [8].

Another study conducted by Croitor et al. indicates that younger patients mostly present with early-stage, low-grade tumors and exhibit lower recurrence and progression rates than their older counterparts [9]. In 2017, Washington et al., using data from the National Cancer Database, found that bladder cancer in younger individuals is usually of lower grade and stage, with a favorable prognosis compared to elderly patients [10]. According to a review of the literature conducted by Alhubaishy et al. in 2021, bladder cancer in young patients is a rare entity and generally has a favorable prognosis and less aggressive tumor characteristics [11]. However, in our study, NMIBC was found in 114 (71.7%) patients, and MIBC was found in 42 (28.3%) patients. Among NMIBC patients, 61 (53.5%) had high-grade tumors. 

In a follow-up study of urinary bladder carcinoma, the 10-year OS rate was 89.55%, while the 10-year CSS rate was 92.24% for young bladder cancer patients [12]. In our study, the five-year survival was approximately 82% for young patients with bladder cancer, 95% for NMIBC patients, and nearly 52% for MIBC patients.

Around 37 (36%) patients had recurrence, and the mean recurrence-free survival was found to be 3.7 years (3.2-4.3 years). According to a study conducted by Woong et al., young patients with urinary bladder carcinoma had a lower recurrence rate (7.1%) compared to adults (38.6%). Recurrence-free survival was 37.7 ± 6.3 months compared to 9.9 ± 2.5 months in adults [13].

The higher prevalence of high-grade tumors, increased recurrence rates, and poorer survival outcomes observed in our study may point toward an aggressive nature of the disease, particularly in this region. This aggressive nature may be attributed to unique environmental factors in North India. For instance, indirect exposure to smoke from the burning of firewood, a common practice during the cold winters in the region, could contribute to the elevated incidence of bladder cancer. Additionally, groundwater consumption, particularly from the Ganges River, has been associated with elevated levels of toxic metals such as lead, arsenic, nickel, chromium, and iron, all of which are recognized carcinogens linked to bladder cancer [6].

However, it is important to emphasize that these hypotheses are speculative in nature. While the association between elevated levels of carcinogens in groundwater and bladder cancer is plausible, direct measurements of water quality or environmental exposures in this cohort were not conducted. As such, we cannot definitively conclude that these factors are responsible for the observed outcomes. Further prospective studies, including direct environmental sampling and more comprehensive exposure assessments, are necessary to validate these potential links.

According to the literature searched to date, this is the largest series of young patients with bladder cancer, taking into account progression and survival, with a maximum follow-up of almost five years. 

Limitations

This study has several important limitations that must be considered when interpreting the results. First, the retrospective nature of the study poses a significant limitation, as it relies on historical data from patient records, which may introduce bias due to incomplete or inconsistent documentation. As a result, the findings cannot establish cause-and-effect relationships but only suggest associations. Another limitation is the single-center design, which means the study reflects the experience of only one institution located in North India. This limits the generalizability of the findings to other regions or populations. Additionally, the study does not include a comparable cohort of older patients, which could have strengthened the assessment of disease aggressiveness and provided a comparison between the two groups. Another key limitation is the absence of multivariate analysis, which restricts the ability to determine the independent impact of various clinical and pathological factors on outcomes such as recurrence and survival, mainly due to small subgroup sizes. Moreover, in the current era of precision medicine, the lack of molecular subtyping or genomic profiling limits the study's ability to offer deeper biological insights or identify targetable alterations.

## Conclusions

Young patients with carcinoma of the urinary bladder exhibit a higher prevalence of high-grade disease and a more aggressive clinical course in North India. While low-grade tumors demonstrate survival outcomes similar to those in adults, high-grade and MIBC are associated with significantly poorer prognoses.
